# Supplementary material for: Caveolin-1 mediates cellular distribution of HER2 and affects trastuzumab binding and therapeutic efficacy
Source: Nat Commun. 2018 Dec 3;9:5137. doi: 10.1038/s41467-018-07608-w (PMC6277446; doi:10.1038/s41467-018-07608-w)
Supplement: Supplementary file 3 — Supplementary Information [file 41467_2018_7608_MOESM3_ESM.pdf]

Caveolin-1 Mediates Cellular Distribution of HER2 and Affects Trastuzumab Binding and Therapeutic Efficacy

Pereira P. M. R. *et al*

Supplementary Figures

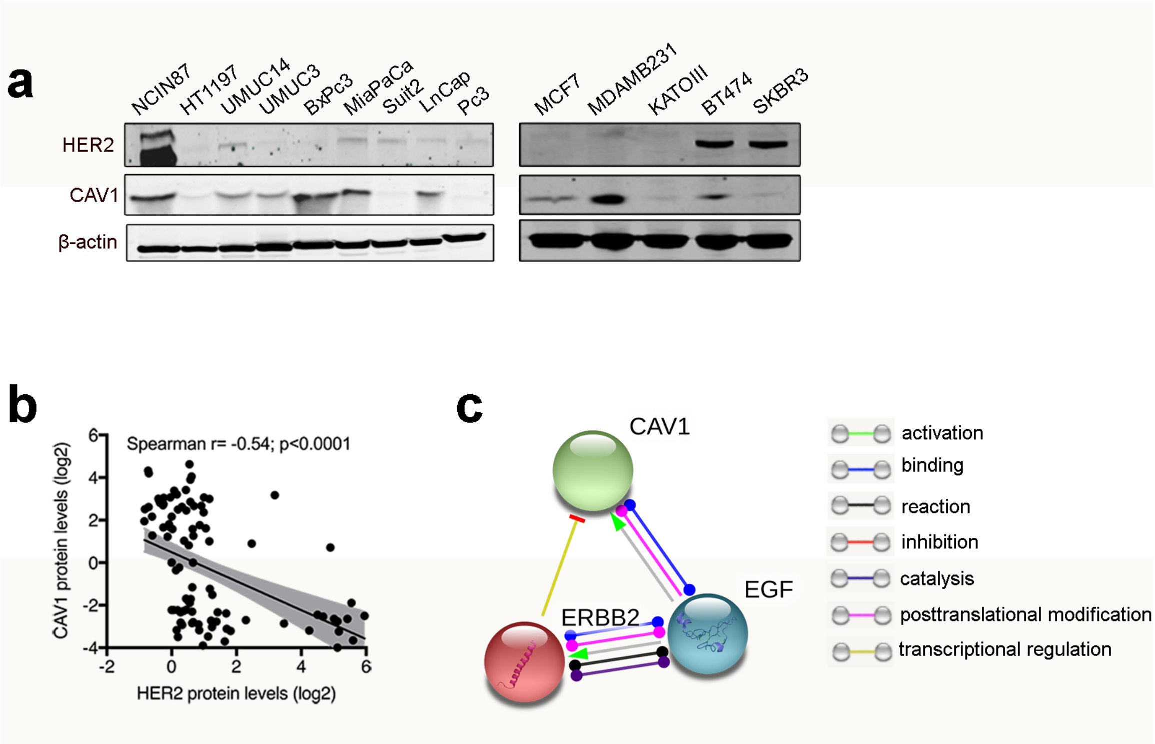

Supplementary Figure 1. a Western blot analysis of HER2 and CAV1 in total lysates of cancer cell lines. **b** Correlation between CAV1 and HER2 proteins in breast, gastric and bladder cancer cell lines from the Cancer Cell Line Encyclopedia. The plot was obtained by excluding cancer cell lines where the log2 expression, of both HER2 and CAV1, was < 0. **c** Interaction network of the HER2–CAV1 protein in STRING database. Source data are provided as a Source Data file.

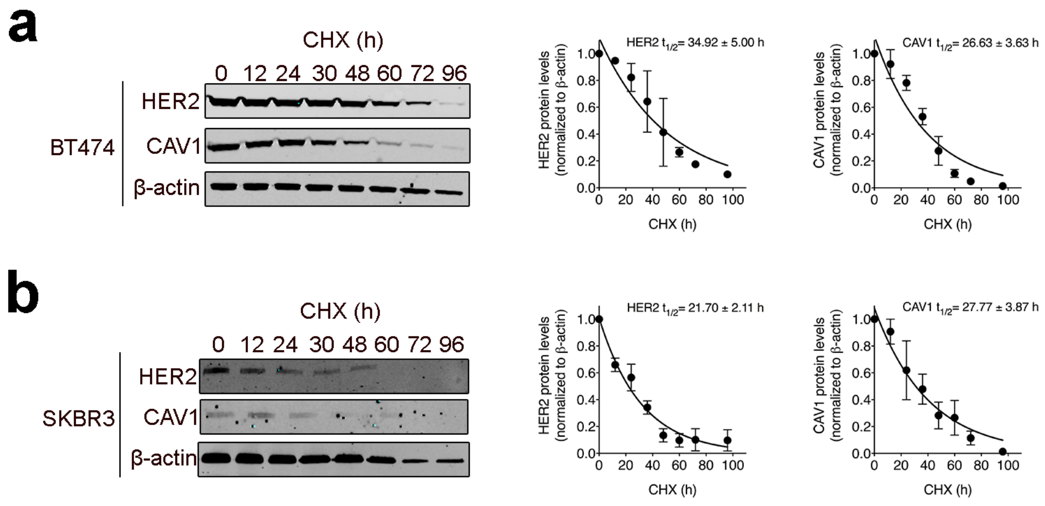

Supplementary Figure 2. Western blot of HER2 and CAV1 in the total lysates of BT474 (**a**) and SKBR3 (**b**) cells after blocking protein synthesis with 80 μg mL^{-1} CHX for 0, 12, 24, 30, 48, 60, 72 and 96h. CHX, cyclohexamide. Half-lives calculated after Western blot analysis. Density of bands on the Western blot was quantified by scanning densitometry with ImageJ software. Half-lives were calculated as the time required for protein decrease to 50% of its initial level (mean ± S.E.M). Source data are provided as a Source Data file.

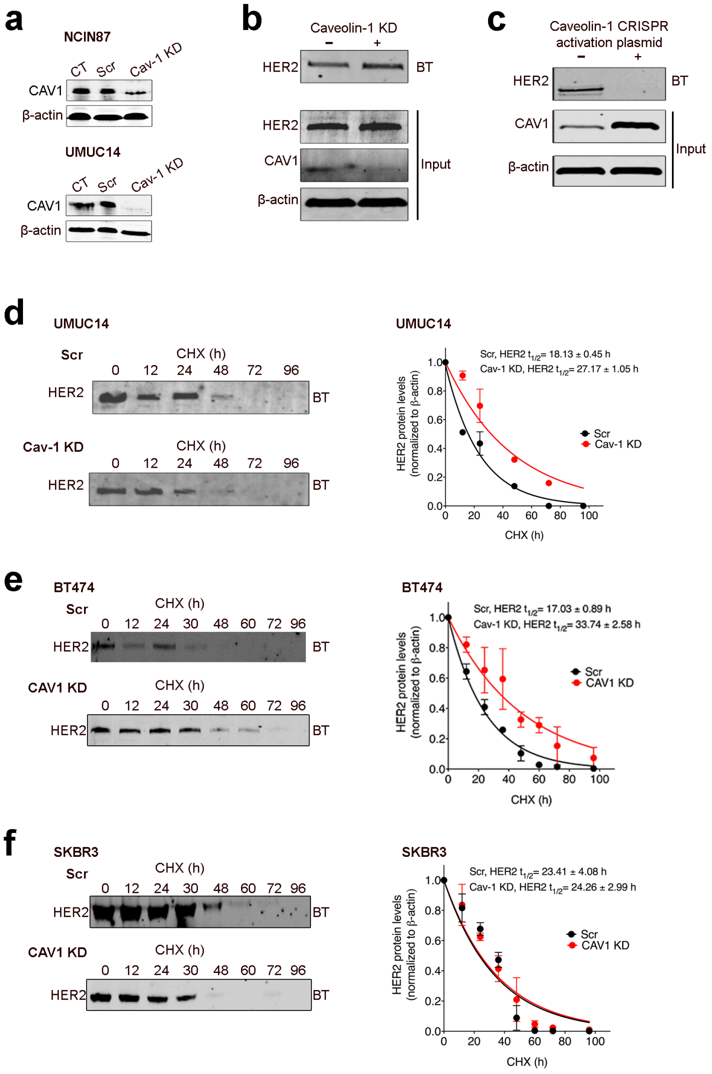

Supplementary Figure 3. a Western blot of CAV1 protein from total extracts of NCIN87 and UMUC14 cancer cells transfected with CAV1 siRNA or scrambled control siRNA constructs for 48 hours. β-actin was used as a loading control. **b** Western blot of biotinylated cell surface–associated HER2 along with HER2 and CAV1 input in the total lysates of UMUC14 cells transfected with CAV1 siRNA or scrambled control siRNA constructs or **c** transfected with CAV1 CRISPR activation plasmid (h) or control CRISPR activation plasmid. BT, biotinylation. **d-f** Western blot of biotinylated cell surface–associated HER2 along with HER2 and CAV1 input in the total lysates of UMUC14, BT474 and SKBR3 cells after transfection with CAV1 siRNA or scrambled control siRNA constructs and blocking protein synthesis with 80 μg mL^{-1} CHX for 0, 12, 24, 30, 48, 60 72 and 96 h. CHX, cyclohexamide. Half-lives of cell surface–associated HER2 calculated after western blot analysis. Density of bands on the Western blots was quantified by scanning densitometry with ImageJ software. Half-lives were calculated as the time required for HER2 protein decrease to 50% of its initial level (mean ± S.E.M). Source data are provided as a Source Data file.

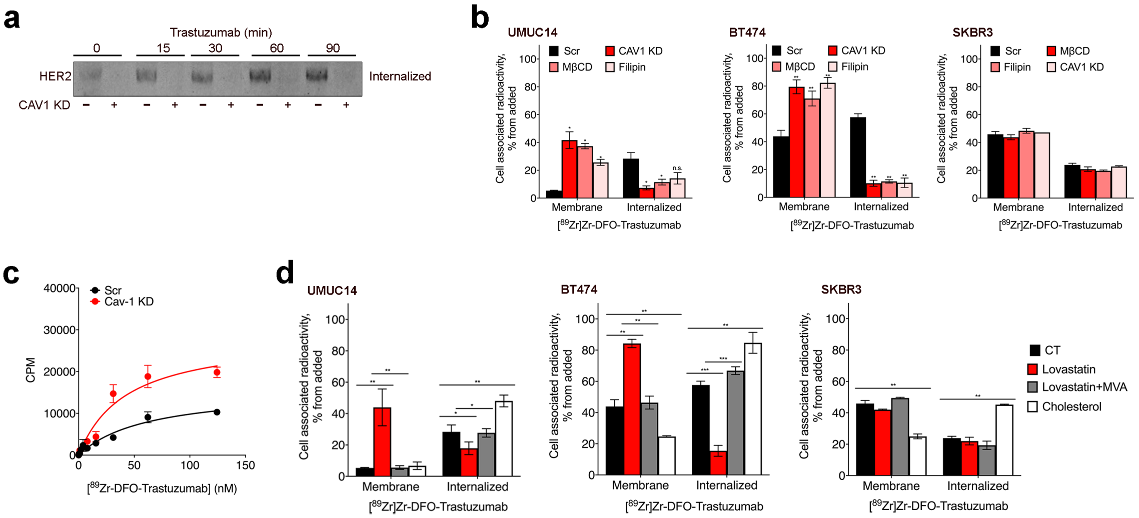

Condition	B_{max} (HERs/cell x 10^5)	K_D (nM)
Scr	2.40	62.02
CAV1 KD	4.34	42.70
Lovastatin	3.50	58.32

Supplementary Figure 4. a CAV1 knockdown decreases the trastuzumab-mediated endocytosis of HER2. Western blot analysis of internalized HER2 on UMUC14 cells transfected with either control or CAV1 siRNA and treated with 1 μM trastuzumab for 0, 15, 30, 60 and 90 min. **b** Membrane-bound and internalized [^{89}Zr]Zr-DFO-trastuzumab before and after CAV1 knockdown or CAV1 depletion with methyl-β-cyclodextrin (MβCD) or filipin in UMUC14, BT474 and SKBR3 cancer cells (bars, n = 4, mean ± S.E.M, *P < 0.05, **P < 0.05,). **c** UMUC14 cells transfected with CAV1 siRNA or scrambled control siRNA constructs were incubated with [^{89}Zr]Zr-DFO-trastuzumab (0 to 125 μM) for 3 h at 4°C. Specifically bound [^{89}Zr]Zr-DFO-trastuzumab plotted *versus* the concentration of [^{89}Zr]Zr-DFO-trastuzumab (upper panel). Specific binding of [^{89}Zr]Zr-DFO-trastuzumab (black and red circles) and nonlinear regression curve fit; data are presented (mean ± S.E.M, n = 3). Binding parameters of [^{89}Zr]Zr-DFO-trastuzumab to UMUC14 cells scrambled control, treated with CAV1 siRNA or with lovastatin (lower panel). **d** Membrane-bound and internalized [^{89}Zr]Zr-DFO-trastuzumab before and after CAV1 depletion with 25 μM of lovastatin for 4 h in UMUC14, BT474 and SKBR3 cancer cells. Increase in membrane-bound trastuzumab is rescued when cells are treated with lovastatin in the presence of 200 μM mevalonic acid (MVA). Treatment of cancer cells with a MβCD solution saturated with cholesterol increases [^{89}Zr]Zr-DFO-trastuzumab internalization (bars, n = 4, mean ± S.E.M, *P < 0.05, **P < 0.01, ***P < 0.001). Source data are provided as a Source Data file.

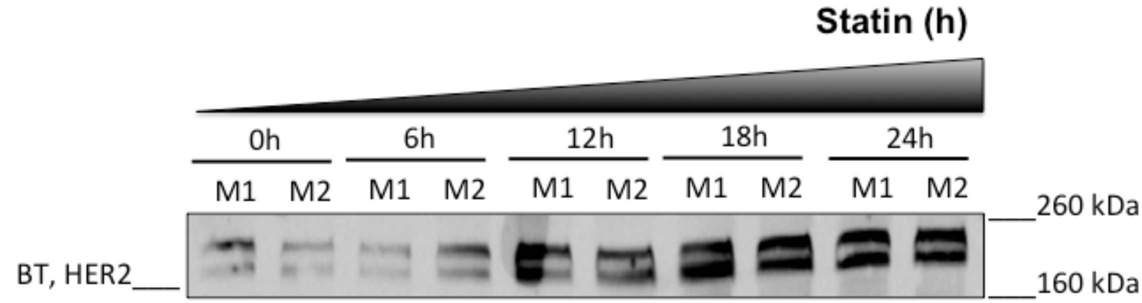

Supplementary Figure 5. Western blot of biotinylated cell surface–associated HER2 in lysates of NCIN87 s.c. tumors from athymic nude mice (n = 2 per group) that were euthanized at different time points t = 0, 12, 18, 24h after treatment with lovastatin. Source data are provided as a Source Data file.

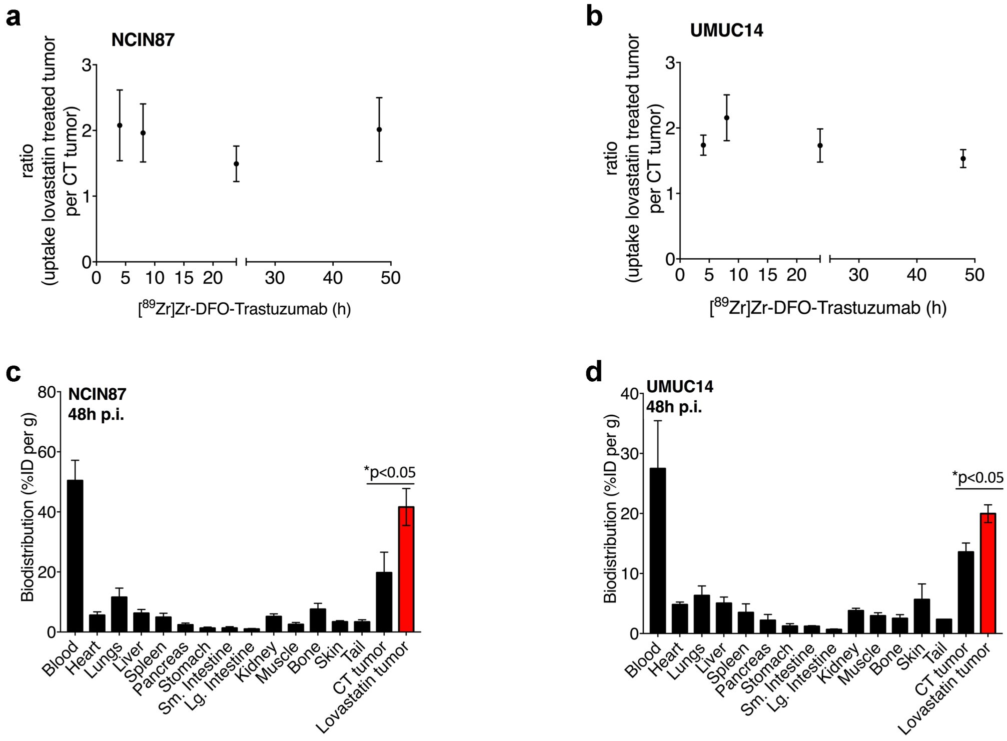

Supplementary Figure 6. Lovastatin treated tumor-to-CT tumor uptake ratios of [^{89}Zr]Zr-DFO-trastuzumab at 4, 8, 24 and 48 h p.i. of [^{89}Zr]Zr-DFO-trastuzumab in athymic nude mice bearing bilateral s.c. gastric (**a**) or bladder (**b**) tumors (n = 4, mean ± S.E.M). Biodistribution data at 48 h p.i. of [^{89}Zr]Zr-DFO-trastuzumab in athymic nude mice bearing bilateral s.c. gastric (**c**) or bladder (**d**) tumors (n = 4, mean ± S.E.M; *, $P < 0.05$). Source data are provided as a Source Data file.

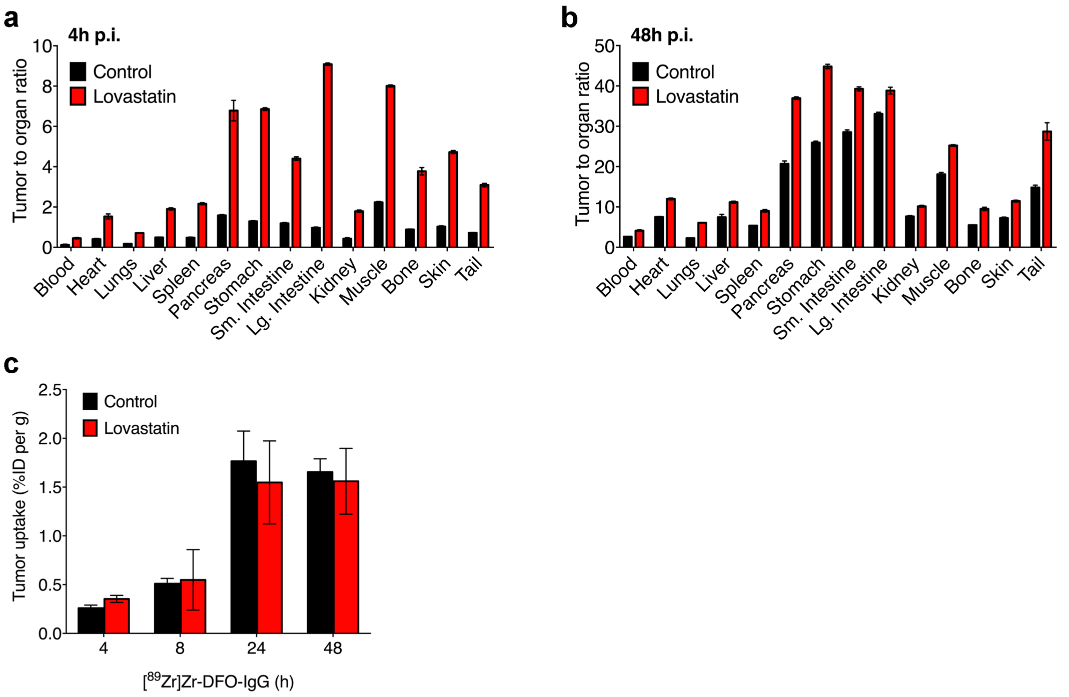

Supplementary Figure 7. Tumor-to-organ ratios at 4 (**a**) and 48 h (**b**) p.i. of [^{89}Zr]Zr-DFO-trastuzumab in athymic nude mice bearing s.c. gastric tumors. Bars, n = 4, mean ± S.E.M. **c** Biodistribution data at 4, 8, 24 and 48 h p.i. of [^{89}Zr]Zr-DFO-IgG in athymic nude mice bearing s.c. NCIN87 gastric tumors. Bars, n = 4, mean ± S.E.M. Source data are provided as a Source Data file.

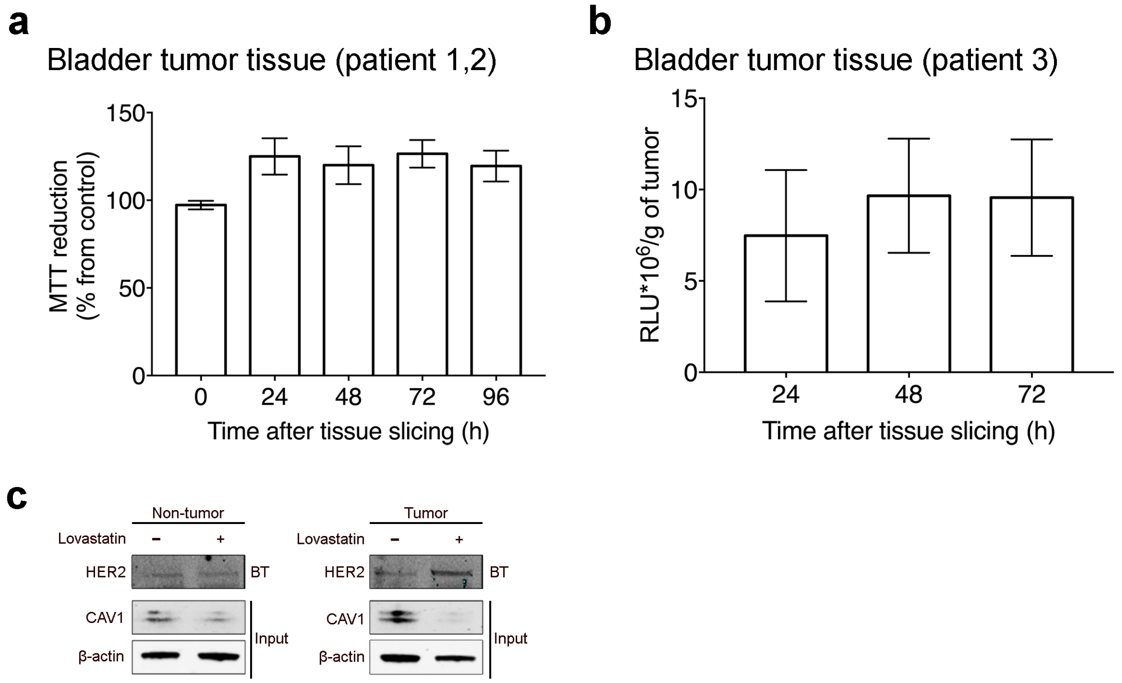

Supplementary Figure 8. Viability of organotypic cultures of bladder tumors as evaluated by (**a**) the MTT and (**b**) CellTiter-Glo assays every 24 h after tissue slicing. Bars, $n = 4$, mean ± S.E.M. RLU, relative light units. **c** Western blot of biotinylated cell surface–associated HER2 and CAV1 input in the total lysates of organotypic cultures of non-tumor and tumor human bladder tissues. Organotypic cultures were incubated with 25 μM of lovastatin twice with an interval of 4 h between each treatment. Tumor lysates were prepared at 3 h after the second dose of lovastatin and analyzed by western blot. Source data are provided as a Source Data file.

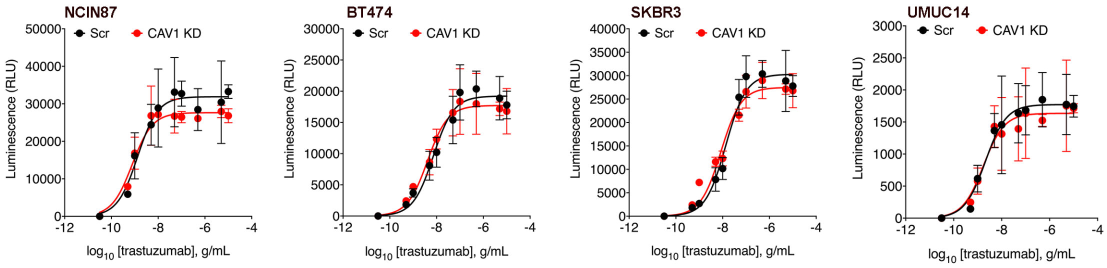

Scr, IC50= 1.3 ng/mL
CAV1 KD, IC50= 0.77 ng/mL

Scr, IC50= 7.2 ng/mL
CAV1 KD, IC50= 4.1 ng/mL

Scr, IC50= 13.7 ng/mL
CAV1 KD, IC50= 10.1 ng/mL

Scr, IC50= 2.1 ng/mL
CAV1 KD, IC50= 1.7 ng/mL

Supplementary Figure 9. ADCC Reporter Bioassay response to trastuzumab using ADCC bioassay effector cells (engineered Jurkat cells stably expressing the FcγRIIIa receptor) and target cells NCIN87, BT474, SKBR3 and UMUC14. Target cells were incubated with a series of concentrations of trastuzumab followed by addition of ADCC bioassay effector Cells. The E:T ratio was 15:1. After 6 hours of induction at 37°C, luciferase assay reagent was added and luminescence was determined using a luminometer. The data were plotted to a 4PL curve using GraphPad Prism. Source data are provided as a Source Data file.

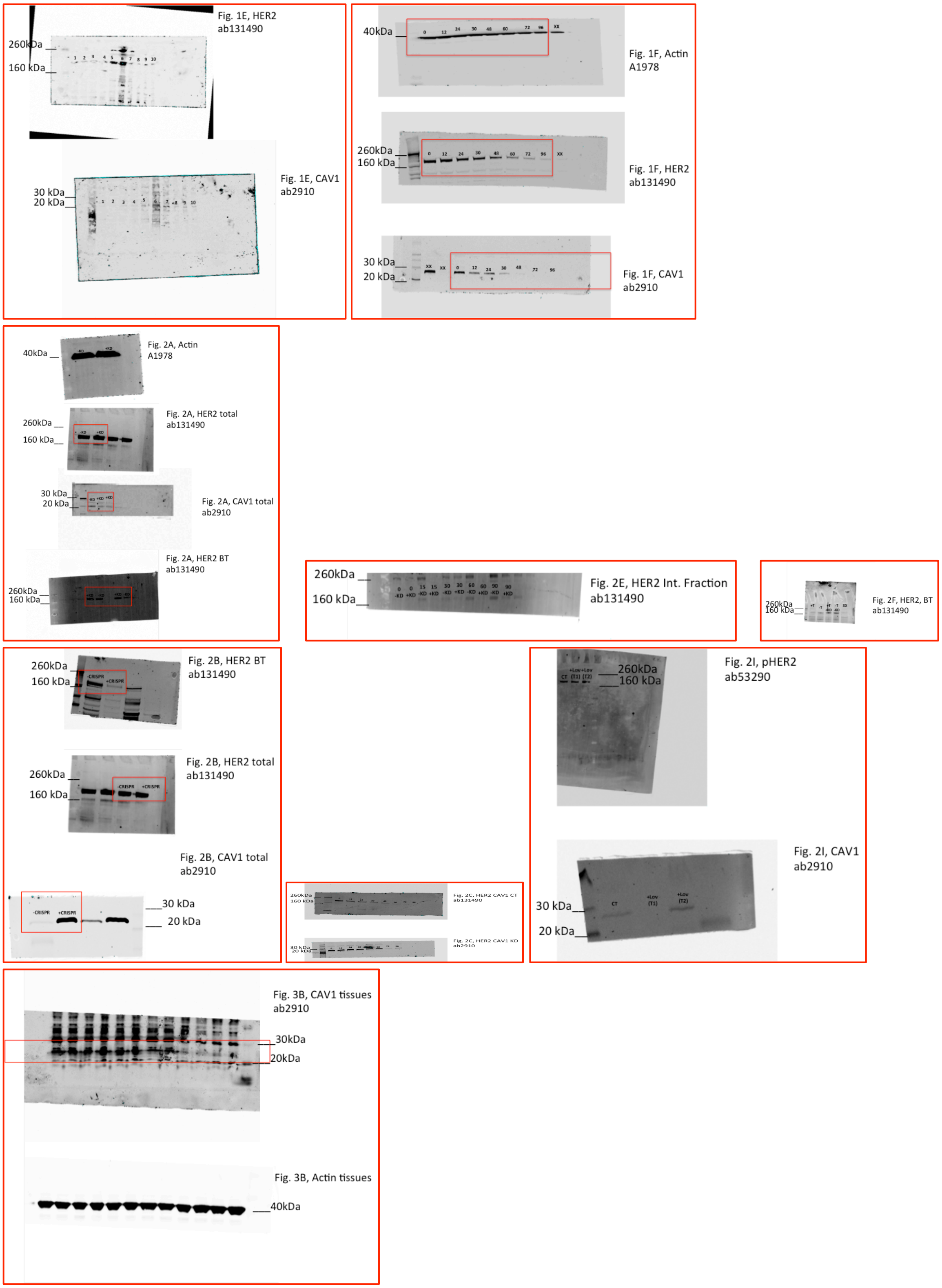

Supplementary Figure 10. Uncropped WB scans of Western Blot images. Source data are provided as a Source Data file.
