# Supplementary material for: Caveolin-1 mediates cellular distribution of HER2 and affects trastuzumab binding and therapeutic efficacy
Source: Nat Commun. 2018 Dec 3;9:5137. doi: 10.1038/s41467-018-07608-w (PMC6277446; doi:10.1038/s41467-018-07608-w)
Supplement: Supplementary file 6 — Description of Additional Supplementary Files [file 41467_2018_7608_MOESM6_ESM.docx]

**Title:** Supplementary Movie 1
**Description:** Live cell imaging of BT474 control cells incubated with Trastuzumab-ICG. BT474 cells were incubated with the conjugate for 1.5 h and live cell imaging was performed during 2 h.

**Title:** Supplementary Movie 2
**Description:** Live cell imaging of BT474 cells pre-treated with 25 μM lovastatin and incubated with Trastuzumab-ICG. BT474 cells were incubated with the conjugate for 1.5 h and live cell imaging was performed during 2 h.
